# Protocatechuic Aldehyde Attenuates UVA-induced Photoaging in Human Dermal Fibroblast Cells by Suppressing MAPKs/AP-1 and NF-κB Signaling Pathways

**DOI:** 10.3390/ijms21134619

**Published:** 2020-06-29

**Authors:** Yuling Ding, Chanipa Jiratchayamaethasakul, Seung-Hong Lee

**Affiliations:** Department of Pharmaceutical Engineering, Soonchunhyang University, Asan 31538, Korea; dingyuling@naver.com (Y.D.); prunchpunch@gmail.com (C.J.)

**Keywords:** ultraviolet A, protocatechuic aldehyde, photoaging, matrix metalloproteinases-1, anti-inflammatory, human dermal fibroblast cells

## Abstract

Ultraviolet radiation (UV) is a major causative factor of DNA damage, inflammatory responses, reactive oxygen species (ROS) generation and a turnover of various cutaneous lesions resulting in skin photoaging. The purpose of this study is to investigate the protective effect of protocatechuic aldehyde (PA), which is a nature-derived compound, against UVA-induced photoaging by using human dermal fibroblast (HDF) cells. In this study, our results indicated that PA significantly reduced the levels of intracellular ROS, nitric oxide (NO), and prostaglandins-E_2_ (PGE_2_) in UVA-irradiated HDF cells. It also inhibited the levels of cyclooxygenase-2 (COX-2) and inducible nitric oxide synthase (iNOS) expression. Besides, PA significantly suppressed the expression of matrix metalloproteinases-1 (MMP-1) and pro-inflammatory cytokines and promoted collagen synthesis in the UVA-irradiated HDF cells. These events occurred through the regulation of activator protein 1 (AP-1), nuclear factor-κB (NF-κB), and p38 signaling pathways in UVA-irradiated HDF cells. Our findings suggest that PA enhances the protective effect of UVA-irradiated photoaging, which is associated with ROS scavenging, anti-wrinkle, and anti-inflammatory activities. Therefore, PA can be a potential candidate for the provision of a protective effect against UVA-stimulated photoaging in the pharmaceutical and cosmeceutical industries.

## 1. Introduction

Skin is the primary protective organ of the integumentary system for humans. It is in direct contact with potentially harmful factors. Thus, it is the most marked tissue that is affected by the aging process [1]. Skin aging is an inevitable process that can be divided into two distinct mechanisms: intrinsic and extrinsic aging [2]. Intrinsic or chronological aging is a natural consequence of physiological changes undergone over the passage of time. Extrinsic aging is a result of exposure to environmental damage, such as pollution, chemicals, cigarette smoking, and ultraviolet (UV) irradiation [3].

UV radiation is a key extrinsic stimulator that mostly results in cumulative skin damage, referred to as photoaging [2]. It is classified based on wavelength into three categories: UVA (320–400 nm), UVB (280–320 nm), and UVC (100–280 nm). Normally, UVC is almost entirely blocked by the ozone layer before reaching the earth’s surface, whereas UVA (approximately, 90–95%) and UVB (5–10%) account for the total UV spectrum found on the earth, which pass through the earth’s atmospheric layers and cause damage to living things [4]. Skin exposure to UV radiation in daily life can induce various physiological effects, such as sunburn, immune suppression, photoaging, skin pigmentation, mutation, DNA and protein denaturation, and the creation of reactive oxygen species (ROS) [5]. Unlike UVB, UVA deeply penetrates the dermis skin layer where the connective tissues and blood vessels are located. Therefore, UVA is a major cause of cell apoptosis and photo-aging in the dermal fibroblast skin.

It has been reported that ROS generation leads to oxidative damage to biological functions, including lipid membrane peroxidation, DNA deterioration, mutation, and apoptosis in the human skin. Moreover, over-accumulation of ROS may stimulate an inflammation response, immune deficiency, and the aging process in resident dermal fibroblasts [4,6].

Furthermore, skin exposure to UV can stimulate age-accelerated enzymes, namely, matrix metalloproteinases (MMPs) such as collagenase (MMP-1) and elastase. An elevation of MMPs is responsible for the degradation of ECM components and other basement membranes. Collagen, elastin, and extracellular matrix (ECM) proteins basically act as a major constructive framework that forms an organized structure and provides the tensile strength for skin [2,7]. Accordingly, the degradation of collagen and elastin is capable of developing into such premature and undesired appearance traits as deep wrinkles, drooping, sagging, and atrophied skin [8]. Therefore, several investigations approaching anti-wrinkling in the skin have focused on the inhibitory activities of collagenase and elastase.

Several investigations also revealed that UV leads to an inflammatory response in the human fibroblast skin resulting in both intrinsic and extrinsic aging [9,10]. It can directly or indirectly trigger several proinflammatory mediators, such as prostaglandin E_2_ (PGE_2_), cyclooxygenase-2 (COX-2), inducible nitric oxide synthase (iNOS), tumor necrosis factor-α (TNF-α), interleukin-1β (IL-1β) and interleukin-6 (IL-6) receptors [5,11]. Notably, these inflammation responses are also associated with skin fibroblast damage, which accelerates the photoaging process induced by UV irradiation.

Protocatechuic aldehyde (PA) is a naturally occurring phenolic aldehyde that can be found in the extracts of various plants and organisms [12,13,14,15]. Previous studies have demonstrated that PA possesses anti-cancer, anti-bacterial, and anti-oxidant effects [15,16,17,18]. In addition, the inhibitory effect of PA on melanogenesis and its inflammatory response via inhibiting the NF-κB signaling pathway has been investigated in previous study [15,19]. Therefore, a lot of evidence indicating bioactivities have proposed that PA can be recognized as a potential bioactive compound. However, there have not been any skin-protective effects of PA against UVA-induced damage reported. In this study, we focused on the protective effects of PA against UVA-irradiation-induced skin photoaging in human dermal fibroblast (HDF) cells.

## 2. Results

### 2.1. Cytotoxicity Effect of PA in HDF Cells

**Figure 1:**
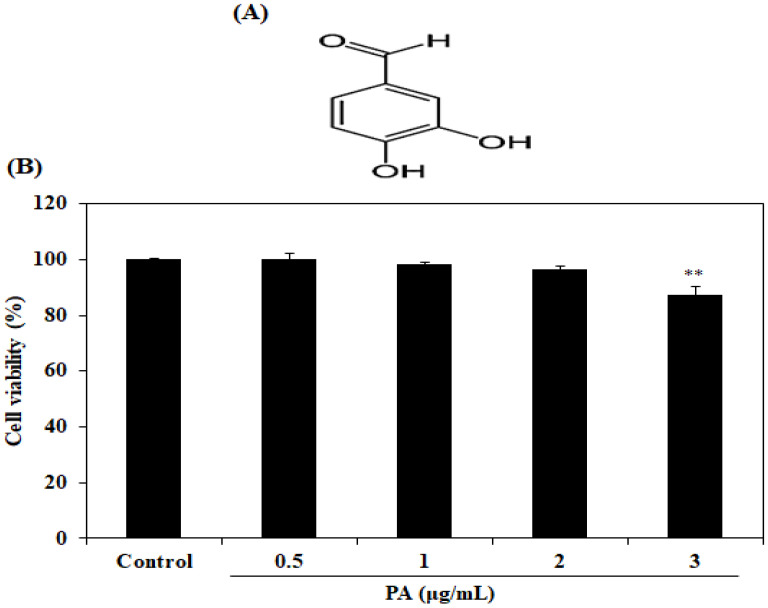
Chemical structure of PA (**A**). Effect of PA on the cell viability of HDF cells treated for 24 h (**B**). The cell viability was measured using an MTT assay. Values are expressed as the mean ± SD in triplicate experiments. ** *p* < 0.01 indicates a significant difference compared to the control group.

The cytotoxicity of PA on HDF cells was investigated to determine the appropriate concentration ranges for cellular experiments. Figure 1B presents the effect of PA at doses of 0, 0.5, 1, 2 and 3 µg/mL on cell viability. The results implied that PA at 3 µg/mL possessed a significant cytotoxicity in the HDF cells. On the other hand, PA doses varying between 0 and 2 µg/mL did not cause major harmful effects on HDF viabilities, and these acceptable doses were selected for investigation for the subsequent experiment.

### 2.2. Effect of PA on Intracellular ROS and Pro-Inflammatory Mediators NO and PGE_2_ Production in UVA-Induced HDF Cells

**Figure 2:**
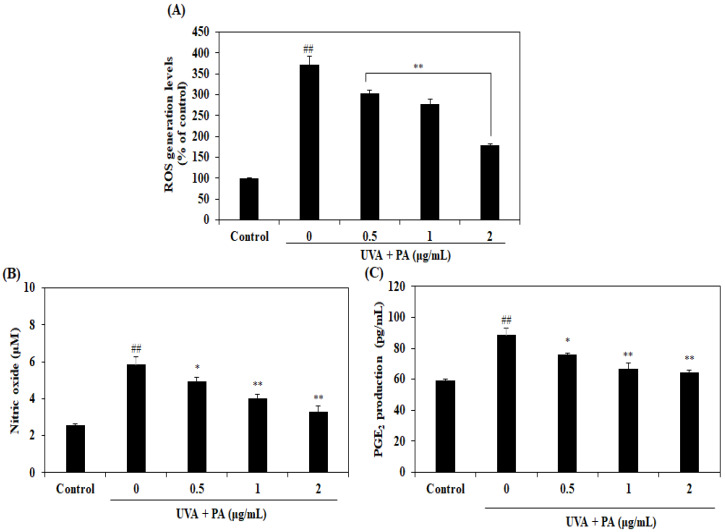
Effects of PA on UVA-irradiated HDF cells. Effects of PA on production of intracellular ROS (**A**) and pro-inflammatory mediators NO (**B**) and PGE_2_ (**C**) in UVA-irradiated HDF cells. Intracellular ROS levels were measured by DCF-DA assay. The amount of NO was measured by Griess reaction Assay. PGE_2_ production was measured by commercial ELISA kits, following the manufacturer’s instructions. Values are expressed as the mean ± SD in triplicate experiments. ^##^ *p* < 0.01 indicates a significant difference compared to the control group. * *p* < 0.05 and ** *p* < 0.01 indicate a significant difference compared to the only UVA-irradiated groups.

On account of it inducing the production of ROS and pro-inflammatory mediators such as NO and PGE_2_, UVA is regarded as one of the major extrinsic factors leading to aging phenomena. Therefore, the present study investigated whether PA may inhibit the generation of UVA-induced ROS and NO. As shown in Figure 2A, the increased production of intracellular ROS induced by UVA irradiation was significantly decreased by PA treatment in a dose-dependent manner. Furthermore, UVA irradiation significantly increased the levels of NO and PGE_2_, while PA treatment remarkably reduced NO and PGE_2_ levels in a dose-dependent manner (Figure 2B and C). These results indicate that PA protects HDF cells from photoaging by scavenging the excess ROS and suppressing the NO and PGE_2_ produced by UVA irradiation.

### 2.3. Effect of PA on COX-2 and iNOS Expression in UVA-Induced HDF Cells

**Figure 3:**
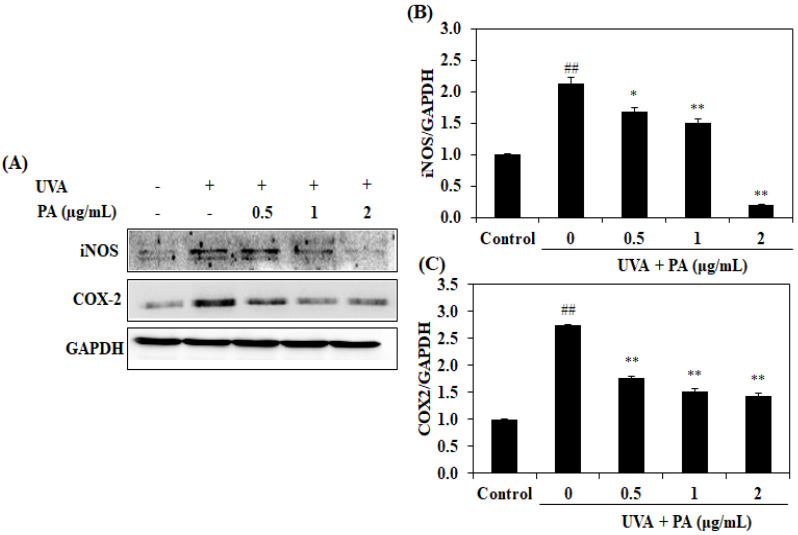
Effects of PA on COX-2 and iNOS expression in UVA-irradiated HDF cells. The iNOS and COX-2 protein expression (**A**) and the relative (**B**) iNOS and (**C**) COX-2 protein expression levels. The levels were compared with GAPDH. The values are expressed as the mean ± SD in triplicate experiments. ^##^ *p* <0.01 indicates a significant difference compared to the control groups. * *p* < 0.05 and ** *p* < 0.01 indicate a significant difference compared to the only UVA-irradiated groups.

We examined the effect of PA on the activation of COX-2 and iNOS because it plays an important role in inducible NO and PGE2 production. Through UVA irradiation, both iNOS and COX-2 expressions were increased compared with the UVA-unirradiated HDF cells. However, PA inhibited UV-induced iNOS and COX-2 expressions in a dose-dependent manner (Figure 3), indicating that PA may inhibit inflammation in HDF cells irradiated with UVA.

### 2.4. Effects of PA on MMP-1 Expression Level and Collagen Synthesis in UVA-Induced HDF Cells

**Figure 4:**
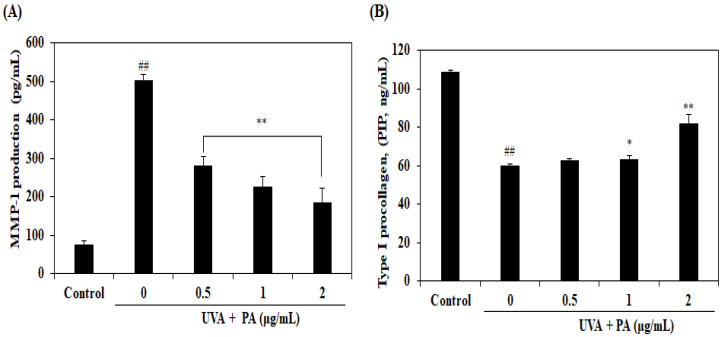
Effects of PA on MMP-1 (**A**) and PIP (**B**) expression in UVA-irradiated HDF cells. The expression levels of MMP-1 and PIP were measured by use of commercial ELISA kits, following the manufacturer’s instructions. The values are expressed as the mean ± SD in triplicate experiments. ^##^ *p* < 0.01 indicates a significant difference compared to the control group and * *p* < 0.05 and ** *p* < 0.01 indicate a significant difference compared to the only UVA-irradiated groups.

MMP-1 is a collagenase that plays a critical role in the degradation of collagen in the skin. Therefore, the MMP-1 expression level in UVA-irradiated HDF cells was investigated. As Figure 4A shows, UVA irradiation significantly stimulated the expression of MMP-1 in UVA-irradiated HDF cells. However, treatment with PA reduced MMP-1 expression level in HDF cells in a dose-dependent manner. Collagen was synthesized as a precursor molecule, procollagen, which contains additional peptide sequences. These sequences were cleaved off during collagen secretion; thus, a number of sequences can indirectly reflect the collagen synthesis level. We determined procollagen type I C-peptide (PIP) so as to investigate the collagen synthesis level. Figure 4B shows that UVA irradiation significantly reduced the collagen level and treatment with PA increased the level of collagen in UVA-irradiated HDF cells in a dose-dependent manner. These results suggest that PA may effectively promote collagen synthesis in HDF cells exposed to UVA by inhibition of MMP-1 expression level.

### 2.5. Effect of PA on Pro-Inflammatory Cytokines Levels in UVA-Induced HDF Cells

**Figure 5:**
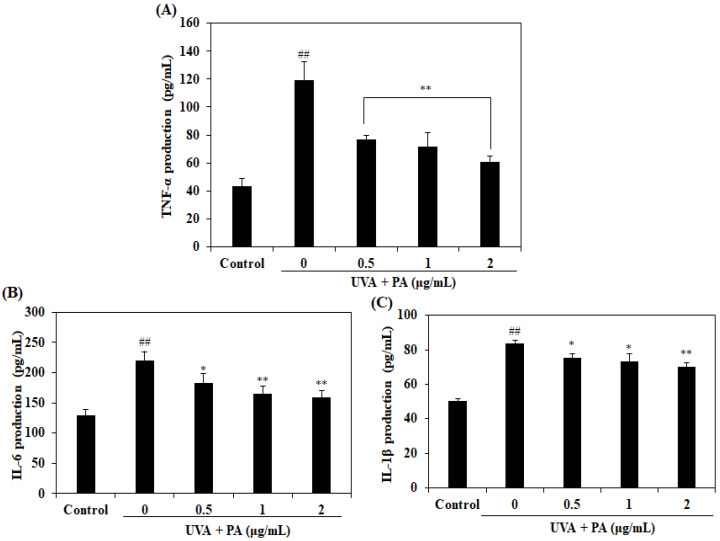
PA inhibits pro-inflammatory cytokine expression in UVA-irradiated HDF cells. Expression of TNF-α in UVA-irradiated HDF cells (**A**); expression of IL-6 in UVA-irradiated HDF cells (**B**); expression of IL-1β in UVA-irradiated HDF cells (**C**). The expression levels of pro-inflammatory cytokines were measured by the use of commercial ELISA kits, following the manufacturer’s instructions. The values are expressed as the mean ± SD in triplicate experiments. ^##^ *p* < 0.01 indicates a significant difference compared to the control group. * *p* < 0.05 and ** *p* < 0.01 indicate a significant difference compared to the only UVA-irradiated groups.

Pro-inflammatory cytokines, such as TNF-α, IL-6, and IL-1β, play an important role in the inflammatory response and also induce expression of MMP-1. Therefore, pro-inflammatory cytokine expression levels in UVA-irradiated HDF cells were investigated. As indicated in Figure 5, the expression levels of pro-inflammatory cytokines, including TNF-α, IL-6, and IL-1β, were significantly increased in UVA-irradiated HDF cells. However, treatment with PA reduced the pro-inflammatory cytokine expression levels in HDF cells in a dose-dependent manner.

### 2.6. Effect of PA on AP-1 and NF-κB Expression in UVA-Induced HDF Cells

**Figure 6:**
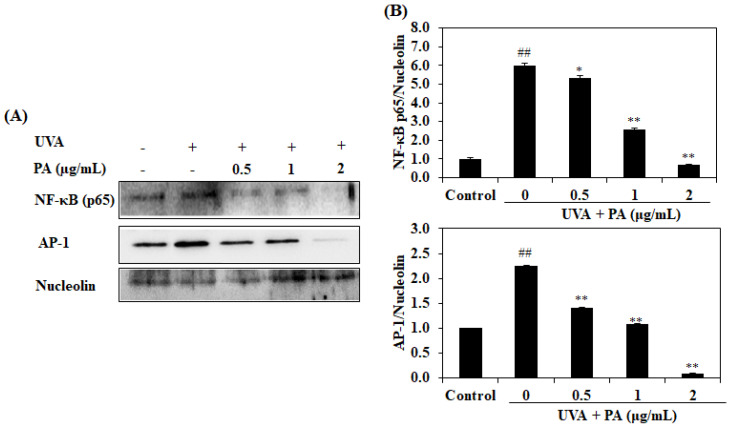
PA inhibits NF-κB activation and AP-1 phosphorylation in UVA-irradiated HDF cells. The inhibitory effects of PA on UVA-irradiated nuclear NF-κB protein expression and AP-1 phosphorylation (**A**) and relative NF-κB expression and AP-1 phosphorylation levels (**B**). The relative NF-κB expression and AP-1 phosphorylation levels were compared with those of nucleolin. The values are expressed as the mean ± SD in triplicate experiments. ^##^ *p* <0.01 indicates a significant difference compared to the control groups. * *p* < 0.05 and ** *p* < 0.01 indicate a significant difference compared to the only UVA-irradiated groups.

MMP-1 and pro-inflammatory cytokine levels are regulated by the AP-1 and NF-κB pathways. The above results indicate the promotive effects of PA on collagen synthesis via inhibition of MMP-1 and pro-inflammatory cytokine expression levels. A western blot analysis was performed to further investigate the role of the AP-1 and NF-κB pathways in PA’s action. As shown in Figure 6, the levels of the activated AP-1 and nuclear NF-κB were significantly increased in UVA-irradiated HDF cells. However, the protein expressions of AP-1 and NF-κB were decreased in the PA-treated HDF cells in a dose-dependent manner. These results indicate that PA suppresses the expression of MMP-1 and pro-inflammatory cytokines through the regulation of the AP-1 and NF-κB pathways.

### 2.7. Effect of PA on Mitogen-Activated Protein Kinase (MAPK) Expression in UVA-Induced HDF Cells

**Figure 7:**
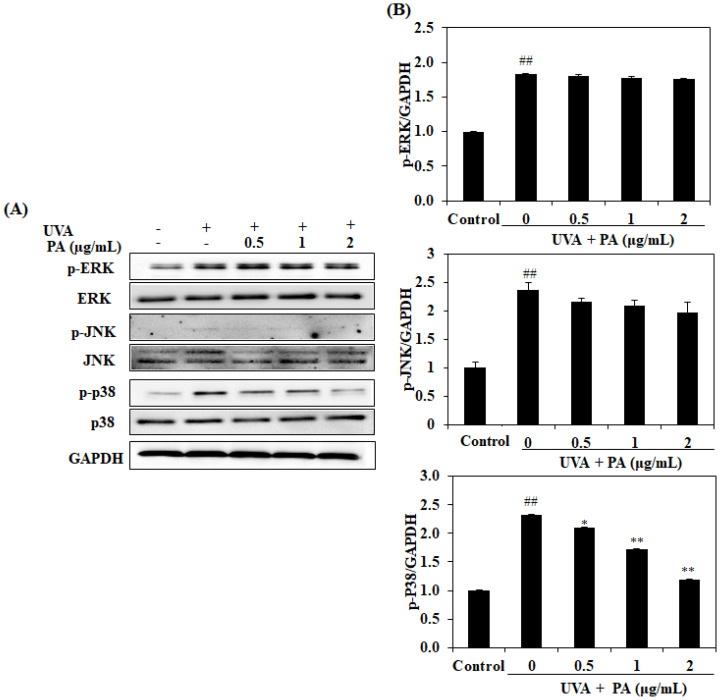
PA suppresses MAPK activation in UVA-irradiated HDF cells. The inhibitory effects of PA on UVA-induced MAPK; ERK, JNK and p38 activation (**A**); and relative activated MAPK levels (**B**). The relative activated MAPK levels were compared with GAPDH. The values are expressed as the mean ± SD in triplicate experiments. ^##^ *p* <0.01 indicates a significant difference compared to the control groups. * *p* < 0.05 and ** *p* < 0.01 indicate a significant difference compared to the only UVA-irradiated groups.

UV irradiation leads to MAPK phosphorylation, which then affects the expression of MMP-1. In order to determine whether PA could inhibit UVA-induced MAPK phosphorylation, we investigated the effects of PA on the phosphorylation of ERK, JNK, and p38 in HDF cells. We found that PA treatment inhibited p38 phosphorylation in UVA-induced HDF cells in a dose-dependent manner, whereas the phosphorylation of ERK and JNK was not significantly affected by PA treatment, as shown in Figure 7. These results suggest that PA-dependent inhibition of p38 phosphorylation may account for the ability of this compound to attenuate MMP-1 expression.

## 3. Discussion

Recently, the ozone layer has been drastically transformed, and its UV ray absorption ability, which fundamentally prevents UV-ray-induced damage, has consistently decreased, causing more intensive UV rays at the Earth’s surface. Therefore, health concerns, such as photo-aging, have become a focus of concern [3,4]. UVA radiation, which penetrates into the deep dermal layer and develops injured skin, can cause major skin damage in the fibroblast and long-term accumulation of UV exposure, resulting in photoaging of skin [20]. Due to awareness of personal health and safety, and toward safer cosmetics free of harmful synthesized substances, attention to using natural compounds as an active ingredient has been increasing in cosmeceutical manufacture [21,22]. In this study, PA, which is a natural phenolic compound, was investigated for whether it provides skin-protective effects against UVA-induced photoaging identified by ROS scavenging, anti-wrinkle, and anti-inflammatory activities.

ROS are free radical molecules containing at least one oxygen atom, which are generated as byproducts of aerobic organisms’ respiratory system, such as superoxide anions (O_2_^−•^), hydroxyl radicals (OH^•^) and singlet oxygen (^1^O_2_). They are highly reactivated molecules, with which unpaired electrons generally initiate chain reactions by donating their single atom or capturing one atom in order to become stable [20]. The UV spectrum is one of the most hostile stimulators and can lead to ROS generation at the molecular and cellular levels. Hence, UVA-induced excess ROS accumulation is very harmful as it contributes to oxidative stress, DNA damage, lipid peroxidation and cell damage as well as skin photoaging [23]. Therefore, ROS scavengers may hold potential to protect against UVA-induced skin aging. In this study, UVA irradiation led to the generation of increased levels of ROS. When the cells were treated with PA, the results demonstrated that ROS levels decreased significantly, compared with the UVA-irradiation-alone group. It was previously reported that PA inhibits hydrogen peroxide-induced ROS generation in normal mouse embryonic liver cells [19]. The previous and present results indicate that PA has intracellular ROS scavenging activity. The present results suggest that PA possesses a protective effect against UVA-induced cutaneous harmful effects, including oxidative stress and photoaging via scavenging intracellular ROS.

It has previously been demonstrated that UVA radiation induced inflammatory responses as a result of the generation of ROS in UVA-exposed skin cells [11,24]. The inflammation responses also accelerate the aging of the skin [10]. Pro-inflammatory mediators, such as NO and PGE_2_, play an important role in the development of inflammation and may serve as a marker of inflammation. The results of the present study clearly demonstrated that the levels of NO and PGE_2_ increased in the HDF cells treated with UVA irradiation alone but were decreased in the UVA-irradiated HDF cells with PA treatment. Upregulation of expression of COX-2 is responsible for PGE2 production, while activation of iNOS expression leads to NO generation [25]. Hence, western blotting further confirmed the occurrence of inflammation following UVA, as increased levels of COX-2 and iNOS protein expression were observed following UVA treatment alone. However, treatment with PA resulted in significantly reduced COX-2 and iNOS protein expression compared with the UVA-irradiation-alone group. Together, these results suggest that PA may inhibit UVA-induced pro-inflammatory mediator production by downregulating COX-2 and iNOS expression.

ROS generation by UVA irradiation can produce oxidative stress and inflammation responses, which subsequently induce the breakdown of collagen by inducing MMP synthesis. MMP-1 is one of the MMP enzymes that majorly break down various types of collagen fibrils (I, II, III, VII, VIII, X, and XI) into generated fragments, resulting in connective tissue destruction and integrity injury of the dermal skin. Hence, the presence of MMP-1 in dermal skin ultimately causes photoaging of the skin, resulting in wrinkle formation, elasticity loss, sagging and atrophy of skin appearance [26,27]. Conversely, type I collagen, which is the most fundamental structure in dermal fibroblast, is synthesized by a precursor molecule that called procollagen. The presence of procollagen in young skin is predominantly higher than in aged skin [28]. The investigated level of PIP leads to collagen synthesis in dermal fibroblasts [29]. Therefore, inhibition of ECM disintegration can facilitate supplementary protection against photoaging of skin induced by UVA. Therefore, the expression levels of MMP-1 and PIP were subsequently determined to investigate the protective effect of PA in UVA-induced photoaging. We observed that treatment with PA significantly inhibited UVA-induced MMP-1 expression and increased the promotion of collagen synthesis. Thus, these results indicated that PA exerted protective effects in UVA-induced photoaging.

Pro-inflammatory cytokines, such as TNF-α, IL-6, and IL-1β, play an important role in the inflammatory response. The UVA-induced pro-inflammatory cytokines are also a powerful stimulator for MMP-1 expression, which can induce the loss of collagen and cutaneous photoaging [30,31]. Therefore, the effects of PA against pro-inflammatory cytokine expression levels in UVA-irradiated HDF cells were further investigated. We observed that UVA irradiation clearly increased the levels of TNF-α, IL-6, and IL-1β production. However, these cytokines were significantly reduced by the treatment with PA. Previous studies have indicated that pro-inflammatory cytokine levels of IL-6 and TNF-α were significantly decreased by PA treatment both in vitro and in vivo [15]. Thus, these results suggest that PA-dependent attenuation of UVA-mediated inflammatory responses may account for the ability of this compound to attenuate MMP-1 expression and photoaging.

Previously, many studies revealed that levels of MMPs are regulated by activated NF-κB, AP-1, and MAPK signaling pathways [23,32,33,34,35]. UV irradiation leads to MAPK phosphorylation, which then directly affects the phosphorylation of nuclear transcription factor AP-1 (c-Jun). This is followed by the up-regulation of MMP expression levels, thus leading to collagen deficiency [3,24]. The MAPK signaling pathway includes ERK, JNK, and p38 MAPK. It was previously reported that UVA irradiation induces the phosphorylation of ERK, JNK, and p38 [36]. To identify whether PA could inhibit UVA-induced MAPK phosphorylation, we investigated the effects of PA on the phosphorylation of ERK, JNK, and p38 in HDF cells. Results demonstrated that treatment with PA significantly inhibited UVA-induced p38 activation. However, no remarkable influences of PA treatment on the expressions of ERK or JNK were observed. These results indicated that PA inhibited the up-regulation of AP-1 expression caused by UV irradiation by inhibiting the p38 signaling pathway.

**Figure 8:**
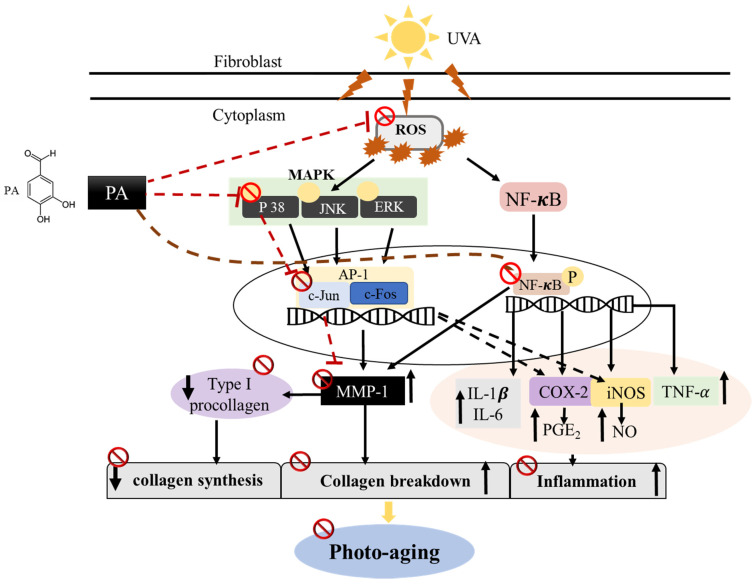
Proposed mechanism for attenuation of UVA-induced photoaging by PA in HDF cells.

NF-κB is a protein complex that plays an important role in immune responses, and its dysregulation is associated with various diseases, such as cancer, inflammation, and aging [3]. NF-κB can be activated by various stimulators, such as ROS and UV irradiation. Activated NF-κB subunits are translocated to the nucleus and induce up-regulation of pro-inflammatory cytokine expression [37]. Furthermore, activation of NF-κB can induce MMP expression [38]. In order to identify the mechanism of PA suppression by MMP-1 and pro-inflammatory cytokine expression, we examined the effects of PA on the phosphorylation of NF-κB in the UVA-induced HDF cells. We observed that treatment with PA significantly inhibited UVA-induced NF-κB activation. These results indicate that PA suppressed the activation of NF-κB, resulting in a down-regulation of pro-inflammatory cytokines and MMP-1 expression. Overall, these results suggest that the mechanism by which PA inhibits UVA-induced photoaging in HDF cells may be related to the regulation of the NF-κB, AP-1, and p38 pathways (Figure 8).

In conclusion, the present study demonstrated that PA reduced the accumulation of UVA-induced ROS, NO and PGE_2_, attenuated MMP-1 expression and inflammation, and enhanced collagen synthesis. PA also inhibits UVA-induced NF-κB, AP-1 and p38 activation, and thereby prevents photoaging in human dermal fibroblasts. To the best of the authors’ knowledge, the present study is the first to report that PA may be beneficial in controlling the development of photoaging and for the protection of UVA-induced damage. Although more extensive studies, including animal and clinical studies, are needed for a thorough understanding of the protective effects of PA on skin photoaging, our results confirm that PA may be a potential active ingredient in anti-photoaging skin treatments.

## 4. Materials and Methods

### 4.1. Materials

Protocatechuic aldehyde (purity; ≥97%, Figure 1A), dimethyl sulfoxide (DMSO), 3-(4-5-dimethylthiazol-2yl)-2-5-diphenylteteazolium bromide (MTT) and fluorescent probe 2′, 7′-dichlorodihydroflurescin diacetate (DCFH-DA) were purchased from Sigma-Aldrich (St. Louis, MO, USA). Dulbecco’s modified Eagle medium (DMEM), phosphate-buffered saline (PBS, 1X), penicillin/streptomycin and fetal bovine serum (FBS) were purchased from Gibco BRL (Life Technologies, Burlington, ON, Canada). The bovine serum albumin (BCA) kit and enhanced chemiluminescence (ECL) reagents were obtained from Thermo Fisher Scientific (Rockford. IL, USA). GAPDH, COX-2, iNOS, nuclear factor-κB (NF-κB), activator protein-1 (AP-1), nucleolin, extracellular signal-regulated kinases (ERK) and phospho-ERK, c-Jun N-terminal kinases (JNK) and phospho-JNK, and p38 and phospho-p38 were purchased from Santa Cruz Biotechnology (Santa Cruz, CA, USA). The anti-rabbit IgG was purchased from Cell Signaling Technology (Beverly, MA, USA). A procollagen type I carboxy-terminal peptide (PIP) ELISA kit was purchased from TaKaRa Bio Inc. (Kusatsu, Japan). Human MMP-1 ELISA kits were purchased from GE Healthcare Life Sciences (Exeter, Devon, UK). PGE_2_, TNF-α, IL-1β and IL-6 ELISA kits were purchased from R&D Systems, Inc. (MN, USA). All other chemicals and reagents used were analytical grade and obtained from commercial sources.

### 4.2. Cell Culture and UVA Irradiation

HDF cell lines obtained from the ATCC (American Type Culture Collection, Manassas, VA, USA) were used to evaluate the UVA protective effect of PA. The HDF cells were cultured in DMEM and F-12 (3:1) mixed medium supplemented with 10% heat-inactivated fetal bovine serum (FBS) and 1% antibiotics (100 unit/mL of penicillin and 100 µg/mL of streptomycin) maintained at 37 °C, under a humidified atmosphere containing 5% CO_2_ in an incubator. UVA irradiation was carried out using a UVA light meter (UV lamp, BLX-LMC, Vilber Lourmat, France) equipped with a fluorescent bulb emitting a 320–400 nm wavelength with a peak at 365 nm. The HDF cells were irradiated at a dose of 20 J/cm^2^ of UVA in PBS. After the radiation, the cells were subsequently replaced with a fresh serum-free medium and incubated until the analysis.

### 4.3. Measurement of Cytotoxicity

The cytotoxicity measurement of PA proceeded at a density of 5 × 10^4^ cells/well in a 24-well plate for 24 h, then treated with PA at four different concentrations—0, 0.5, 1, 2 and 3 μg/mL—for another 24 h. The cell viability of HDFs was determined by using a colorimetric MTT assay as described by Ding et al. [39]. Briefly, 100 µL of MTT stock solution (2 mg/mL in PBS buffer) was added to each well and then incubated for 3 h. After the incubation, each of the mixture mediums was discarded and 300 µL of 100% DMSO was added to dissolve an insoluble formazan. The absorbance was measured at 540 nm using a microplate reader (Sunrise TW, Tecan Trading AG, Mannedorf, Switzerland).

### 4.4. Determination of Intracellular ROS Levels

Intracellular ROS levels were measured using the DCF-DA assay [40]. The cells were irradiated with UVA (20 J/cm^2^) and incubated for 1 h at 37 °C. After incubation, the cells were treated with or without the indicated concentrations of PA for 1 h. Subsequently, DCF-DA (0.5 mg/mL) was added to the HDF cells and incubated for 30 min. Finally, DCF-DA was introduced to the cells, and 2′,7′-dichlorodihydrofluorescein fluorescence was detected at an excitation wavelength of 485 nm and an emission wavelength of 535 nm, using a fluorescence plate reader (Perkin Elmer Inc., Waltham, MA, USA).

### 4.5. Determination of Nitric Oxide (NO) Production

NO production in supernatants was measured using the Griess reagent [41]. Briefly, the cells were irradiated with UVA at 20 J/cm^2^ and, after 1 h, the cells were treated with or without the indicated concentrations of PA for 24 h. Next, the supernatant was collected to measure the nitrite accumulated in a culture medium. A quantity of 100 μL of supernatant was mixed with an equal volume (1:1) of Griess reagent (1% sulfanilamide and 0.1% N-(1-naphthyl)-ethylenediamine and 5% phosphoric acid). The mixture was incubated at room temperature for 10 min, and the absorbance at 540 nm was measured using a microplate reader. Sodium nitrite (NaNO_2_) was used to produce a standard curve of nitrite concentrations (µM).

### 4.6. Enzyme-Linked Immunosorbent Assay (ELISA) Determination of MMP-1 and Type I Procollagen Production

HDF cells were irradiated with UVA (20 J/cm^2^) and incubated for 1 h at 37 °C. The cells were then treated with or without the indicated concentrations of PA for 24 h. The cell culture medium was collected and used to assess the production of pro-inflammatory mediators or cytokines (PGE_2_, TNF-α, IL-1β, and IL-6), MMP-1 expression level and collagen synthesis. The amount of the pro-inflammatory cytokines, MMP-1 and procollagen type I C-peptide (PIP), which reflect the level of collagen synthesis, were measured by use of commercial ELISA kits following the manufacturer’s instructions.

### 4.7. Western Blot Analysis

The cells were irradiated with UVA and treated with PA at the indicated concentrations (0–2 μg/mL). Cells were washed with cold PBS twice and harvested. Next, proteins were extracted with the PRO-PREP protein extraction kit (iNtRON Biotechnology, Sungnam, Korea). The protein contents of total cell lysate were measured by using the BCA^TM^ protein assay kit (Sigma-Aldrich, St. Louis, MO, USA). The samples containing equal protein contents were loaded on 15% sodium dodecyl sulfate–polyacrylamide gel electrophoresis (SDS-PAGE) and separated by electrophoresis, then transferred to a nitrocellulose membrane. At the blocking step, the membrane was blocked in 5% nonfat dry milk in TBST (25 mM Tris–HCl, 137 mM NaCl, 2.65 mM KCl, 0.05% Tween 20, pH 7.4) for 2 h. The 1:1000 dilutions of different primary antibodies in 5% nonfat dry milk solutions were individually incubated overnight at 4 °C. Thereafter, the membranes were washed and incubated with 1:3000 dilution secondary antibodies for 2 h at room temperature. All protein bands were developed using an ECL western blotting detection kit. The relative expressions of all proteins were analyzed by using the Image J program (Wayne Rasband, National Institutes of Health, Bethesda, MD, USA).

### 4.8. Statistical Analysis

All experiments were carried out in triplicate and data were described as the mean ± standard deviation (SD). Statistical analysis was done using the one-way analysis of variance (ANOVA) complemented by the Duncan’s multiple range test. The level of statistical significance was considered at *p* < 0.05. The degrees of significance are indicated as follows: * *p* <0.05, ** *p* <0.01, ^##^ *p* < 0.01.
